# Retinoic Acid and Germ Cell Development in the Ovary and Testis

**DOI:** 10.3390/biom9120775

**Published:** 2019-11-24

**Authors:** Tsutomu Endo, Maria M. Mikedis, Peter K. Nicholls, David C. Page, Dirk G. de Rooij

**Affiliations:** 1 Immunology Frontier Research Center, Research Institute for Microbial Diseases, Osaka University, 3-1 Yamadaoka, Suita 565-0871, Japan; 2 Whitehead Institute, Cambridge, MA 02142, USA; 3 Department of Biology, Massachusetts Institute of Technology, Cambridge, MA 02139, USA; 4 Howard Hughes Medical Institute, Whitehead Institute, Cambridge, MA 02142, USA; 5 Reproductive Biology Group, Division of Developmental Biology, Department of Biology, Faculty of Science, Utrecht University, 3584 CH Utrecht, The Netherlands; 6 Center for Reproductive Medicine, Academic Medical Center, University of Amsterdam, 1105 AZ Amsterdam, The Netherlands

**Keywords:** germ cells, retinoic acid, meiosis, ovary, spermatogenesis, testis

## Abstract

Retinoic acid (RA), a derivative of vitamin A, is critical for the production of oocytes and sperm in mammals. These gametes derive from primordial germ cells, which colonize the nascent gonad, and later undertake sexual differentiation to produce oocytes or sperm. During fetal development, germ cells in the ovary initiate meiosis in response to RA, whereas those in the testis do not yet initiate meiosis, as they are insulated from RA, and undergo cell cycle arrest. After birth, male germ cells resume proliferation and undergo a transition to spermatogonia, which are destined to develop into haploid spermatozoa via spermatogenesis. Recent findings indicate that RA levels change periodically in adult testes to direct not only meiotic initiation, but also other key developmental transitions to ensure that spermatogenesis is precisely organized for the prodigious output of sperm. This review focuses on how female and male germ cells develop in the ovary and testis, respectively, and the role of RA in this process.

## 1. Introduction

Mammalian oocytes and sperm derive from the same embryonic precursor cells, called primordial germ cells (PGCs). In development, PGCs migrate to the somatic gonad, where they undertake gametogenesis to ultimately produce oocytes or sperm, depending on whether they are in an ovary (female) or a testis (male). In mice, differences between the somatic cellular composition of ovaries and testes are microscopically evident by embryonic day (E) 12.5 [1,2,3]. Germ cells, however, remain morphologically indistinguishable between the sexes until E13.5 [4,5]. Subsequently, female germ cells enter meiotic prophase I and begin to differentiate as oocytes, whereas male germ cells remain mitotically active, and later arrest in the G0/G1 phase of the mitotic cell cycle [4,5].

In the 1970s, Byskov and Saxen [6] suggested that a “meiosis inducing substance”, present in the embryonic ovary, is required for germ cells to initiate meiotic prophase. Recent studies find that retinoic acid (RA) is such a meiosis-inducing substance, produced by the somatic cells of the gonad and mesonephros [7,8,9]. RA is generated from dietary vitamin A (also known as retinol) by a series of oxidative reactions. Local levels of RA are regulated by retinaldehyde dehydrogenases, which catalyze the last step of RA synthesis, and by a cytochrome p450 enzyme (CYP26B1), which degrades RA [10,11] (reviewed in [12,13]). This metabolism of RA regulates whether female and male germ cells initiate meiosis in the fetal ovary, or the adult testis, respectively.

Male germ cells, which are arrested in G0/G1 phase of the cell cycle in the fetal testis, later resume proliferation and undergo a transition to spermatogonia after birth [14,15]. Spermatogonia, which include germline stem cells, undergo an elaborately organized process to give rise to specialized haploid gametes, called spermatozoa [16]. The complete process of germ cell development, from spermatogonia to spermatozoa, is called spermatogenesis. Within the testis, several developmental transitions of spermatogenesis, including spermatogonial differentiation and meiotic initiation, occur in close physical and temporal proximity. Over several decades, pharmacological and genetic studies have revealed that these key transitions are strictly regulated by RA [17,18,19]. In this review, we focus on how germ cell development is coordinated in the ovary and testis, and the instructive role of RA in this process.

## 2. Germ Cell Development in the Fetal Gonad

Shortly after entry to the gonads, germ cells acquire the competence for meiotic initiation and sexual differentiation in the fetal gonad. Whether germ cells initiate meiosis or continue in a mitotic cell cycle is determined by their gonadal environment, rather than their sex chromosome constitution (reviewed in [20]). Germ cells in the fetal ovary are exposed to RA and initiate meiosis, whereas those in the fetal testis are sequestered from RA signaling and do not initiate meiosis until after birth.

### 2.1. Formation of the Gonad and Migration of PGCs to the Gonad

**Figure 1:**
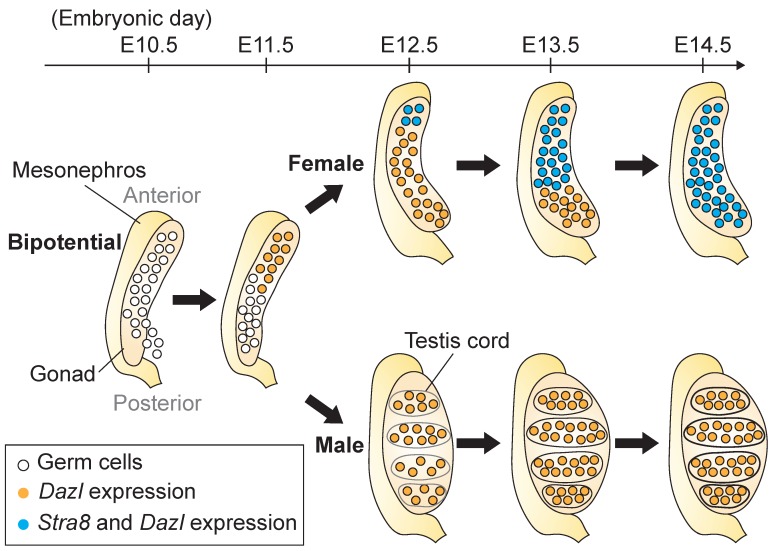
Anterior-to-posterior wave of *Dazl* and *Stra8* expression from E10.5 to E14.5 in mouse fetal gonads. Germ cells are shown in circles, with cells expressing *Dazl* shown in orange, and cells expressing *Stra8* and *Dazl* shown in blue. After gonadal colonization, germ cells continue to proliferate until E13.5 [22]. In the fetal mouse testis, germ cells become enclosed by somatic cells, with testis cords formed between E12.5 to E14.0 [14,23].

In mammals, both the ovary and testis derive from a common precursor structure, the bipotential gonad (Figure 1) [21]. The development of the bipotential gonad involves two simultaneously occurring processes. The coelomic epithelium develops into a thickened, multilayer structure, known as the genital ridge. This differentiation initiates at the coelomic epithelium’s anterior end and extends posteriorly [3,21]. In mice, the development of the bipotential gonad begins at around E10.0 and continues until E11.5–E12.0 [1,2,3]. Thereafter, the gonad’s somatic cells undergo sexual differentiation [1,2,3].

Meanwhile, PGCs, the precursors of sperm and eggs, are induced early in embryogenesis, and later migrate to the developing genital ridge [22]. Throughout their migration, PGCs maintain a transcriptional program of developmentally uncommitted cells, marked by the expression of both naïve and general pluripotency factors [24,25,26]. Upon colonization of the nascent gonad, human and mouse PGCs induce a set of germ cell factors, including evolutionarily conserved markers of germ granules [24]. After their arrival in the gonad, PGCs subsequently down-regulate the expression of pluripotency factors, and lose the capacity to give rise to pluripotent cell lines (known an embryonic germ [EG] cells) and teratomas, a tumor arising from pluripotent cells [24,27,28]. This transition, broadly conserved among vertebrates, serves to restrict the developmental potential of the mammalian germ line, a process termed germ cell determination [24].

### 2.2. Initiation of Gametogenesis and Meiotic Entry

**Figure 2:**
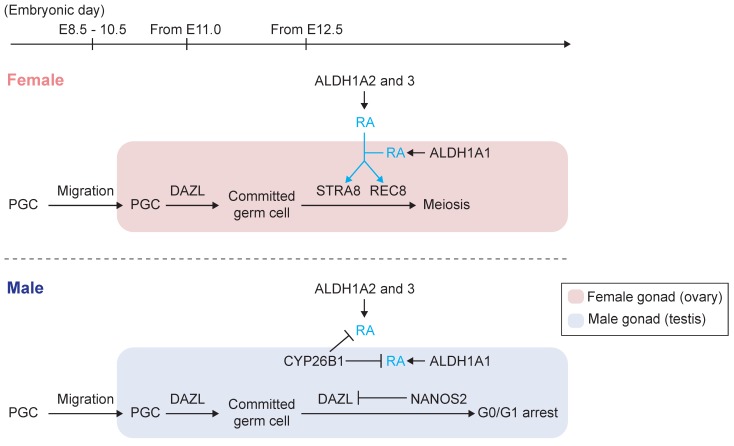
Diagram of germ cell development in mouse fetal gonads of both sexes. Red box: female gonad (ovary). Blue box: male gonad (testis). DAZL, STRA8, REC8, and NANOS2 are expressed in germ cells. ALDH1A1 and CYP26B1 are expressed in fetal gonads. ALDH1A2 and ALDH1A3 are expressed outside the gonads.

Once determined, germ cells are poised to initiate meiosis, as well as undertake male or female differentiation [29,30]. The transition of PGCs to committed germ cells represents a critical transformation of the germ line to a sexually competent state [31], and is induced by extrinsic signals from the genital ridge [32]. One of the genes induced at PGC colonization in mice and humans is *Dazl* [24], which encodes an evolutionarily conserved and germ-cell-specific RNA-binding protein (Figure 2) [33]. In *Dazl*-null mouse embryos, PGCs arrive at the gonad, but fail to restrict their developmental potential; instead, these cells remain proliferative, continue to express pluripotency factors, retain the capacity for the derivation of pluripotent EG cells until at least E15.5, and fail to initiate meiosis or embark upon spermatogenesis or oogenesis in the fetal testis or ovary, respectively [24,31,34,35]. Consistent with the failure to restrict germline potential, *Dazl*-deficient mice and pigs develop spontaneous teratomas at an elevated frequency [24]. Thus, *Dazl* is necessary for the germ line to undertake a restriction of potential, and for the competence to undertake gametogenesis, defined as the capacity to initiate meiosis and sexual differentiation [31].

On expression of *DAZL*, germ cells acquire the competence to interpret RA as a meiosis-inducing signal (Figure 2) [7,8,35]. RA induces germ cells to express both *Stra8* (*Stimulated by Retinoic acid gene 8*), a gene required for meiotic initiation [36], and *Rec8*, a gene required for meiotic progression [37,38] (Figure 1 and Figure 2) [39,40]. These two factors are independently activated by RA (Figure 2) [39,41] and precede the expression of other meiotic markers, such as *Dmc1*, *Sycp3*, and the phosphorylation of histone H2AX (γH2AX), which is a marker of meiotic double strand breaks [26,40,42,43].

Like the preceding differentiation of the somatic cells, many aspects of germline development occur in an anterior-to-posterior (A-P) wave along the length of the gonad [26,32,40,42,44]. At E11.5, newly arrived PGCs show a gradient of *Dazl* expression, which is highest in the anterior portion of the gonad and low or absent in the posterior portion (Figure 1) [32].

### 2.3. Stra8 and Its Inducer, RA, Regulate Meiotic Initiation in the Fetal Ovary

*Stra8* is highly expressed in germ cells of both sexes at meiotic initiation, before quickly turning off early in meiosis [18,36,40]. *Stra8* expression in ovarian germ cells begins at E12.5 and progresses in a subsequent A-P wave, such that the expression of *Stra8* and other meiotic markers is heterogeneous across the population of germ cells (Figure 1) [26,39,40]. In the fetal ovary, *Stra8* is first detected within one day prior to when the characteristically condensed chromatin of meiotic germ cells can be observed (Figure 1 and Figure 2) [40]. In mice of the C57BL/6 genetic background, *Stra8*-null ovarian germ cells do not undergo meiotic DNA replication [36], nor do they robustly express meiotic factors or begin the chromosomal events of meiotic prophase I [36,41]; thus, *Stra8* is necessary for meiotic initiation in mice. STRA8 is a transcriptional activator that binds to the promoters and enhances the expression of thousands of genes, including meiotic prophase I genes, G1-S cell-cycle genes, and factors that specifically inhibit the mitotic program [45]. In fetal testes, male germ cells do not express *Stra8* (Figure 1 and Figure 2) [40]. Instead, *Stra8* is first expressed much later in germ cells of postnatal testes, when they undergo differentiation [46,47,48].

A potential link between RA and meiotic initiation was initially provided by in vivo studies of the *Stra8* gene [36,40], which was first identified as an RA-inducible gene in embryonal carcinoma cells and embryonic stem cells in vitro [46]. In fetal ovaries, all-*trans* RA robustly induces Stra8 expression and thereby meiotic initiation (Figure 2) [7,8]. Exogenous all-*trans* RA is sufficient to induce ectopic *Stra8* expression, and for the precocious initiation of meiosis in fetal testes [7,8]. Later work provided direct evidence for RA’s role in meiotic initiation—in the ovaries of vitamin A-deficient rat embryos, *Stra8* is not robustly activated, and germ cells fail to enter meiosis [49]. Thus, RA can induce meiotic initiation in both female and male germ cells of the fetal gonad.

Two families of nuclear hormone receptors, known as RA receptors (RARs) and retinoid X receptors (RXRs), bind RA. RARs bind both all-*trans* and 9-*cis* RA stereoisoforms, while RXRs bind only 9-*cis* RA [50]. RXRs can also bind other ligands that are not derived from RA [51] (reviewed in [52,53]), but it is unclear whether these ligands contribute to meiotic initiation. RARs and RXRs interact to form heterodimers that bind to RA response elements (RAREs) in the regulatory regions of target genes [54]. RXRs can also heterodimerize with other nuclear hormone receptors (reviewed in [55]), but whether these interactions promote meiotic initiation is not yet known. RARs and RXRs each have three isotypes (RARα, RARβ, and RARγ, and RXRα, RXRβ, and RXRγ), and each exhibits overlapping expression and functional redundancy in many tissues (reviewed in [54,56,57]). Both RAR and RXR isotypes are expressed in the gonads of each sex [8,58,59,60,61]. In embryonic ovaries, RARs are readily detected in germ cells but are expressed at very low levels, if at all, in somatic cells [8,58,62], while RXRs are found in both somatic and germ cells [8,62]. The promoter of the *Stra8* gene contains two putative RAREs, suggesting that RA may directly up-regulate *Stra8* transcription by binding to RAR/RXR heterodimers engaged at the *Stra8* promoter [46,63]. Indeed, antagonists of the RARs diminish or block *Stra8* expression, while exogenous RA induces *Stra8* expression in the fetal ovary [7,8].

### 2.4. Source of RA in the Fetal Ovary

RA originating from both the somatic cells of the fetal ovary and mesonephros likely contribute to meiotic initiation (Figure 2) [8,9]. Initial studies identified the mesonephros as a robust source of RA, as these cells strongly expressed a *lacZ* reporter transgene under the control of an RARE [8]. Weaker *RARE*-*lacZ* signal was detected in the fetal gonad, with the strongest gonadal signal detected at the anterior end [8]. The mesonephros expresses two RA-synthesizing enzymes (Figure 2), *aldehyde dehydrogenase 1A2* (*Aldh1a2*) [8] and *Aldh1a3* [64]. Upon deletion of *Aldh1a2* or both *Aldh1a2* and *Aldh1a3*, the mesonephros fails to produce RA, as evidenced by the loss of *RARE*-*lacZ* signal in transgenic mice [65]. At the same time, the ovarian germ cells from these mutant embryos express *Stra8* and initiate meiosis [65]. Therefore, mesonephros-derived RA is not strictly required for meiotic initiation.

Based on these findings, some have proposed that RA itself is not required for meiotic initiation in the ovary [65]. However, subsequent work demonstrated that germ cells from cultured fetal ovaries initiate meiosis in the absence of the mesonephros, suggesting that an alternative source of RA—such as the fetal ovary—is sufficient for meiotic initiation [66]. Additional studies indicated that the somatic cells of the fetal gonad express *Aldh1a1* and therefore produce RA (Figure 2) [9,66,67]. Further, genetic deletion of *Aldh1a1* decreases RA levels in the fetal ovary [9]. While *Aldh1a1*-deficient fetal ovaries initially exhibit reduced expression of *Stra8* and other genes that are usually upregulated at meiotic initiation, these meiotic factors are expressed at similar levels one day later, suggesting that RA derived from the mesonephros allows the germ cells to initiate meiosis and overcome the earlier delay [9]. Consistent with this recovery, *Aldh1a1*-null female mice are fertile [68]. Therefore, RA derived from the fetal ovary via *Aldh1a1* regulates the timing of meiotic initiation, but is not strictly required. At the same time, *Aldh1a1* provides sufficient RA to initiate meiosis in the ovaries of *Aldh1a2*-null; *Aldh1a3*-null embryos.

That *Aldh1a1* is redundant for meiotic initiation may be accounted for by its inverse expression in response to RA levels. In fetal testes lacking *Cyp26b1*, endogenous RA levels are elevated, and *Aldh1a1* expression is greatly reduced, suggesting a negative feedback loop between RA signaling and *Aldh1a1* expression [9]. Therefore, the elimination of mesonephros-derived RA by deletion of *Aldh1a2* and *Aldh1a3* may cause an increase in *Aldh1a1* expression in the gonad, raising RA levels in the fetal ovary [9]. In the embryonic ovary, RA produced by both the mesonephros and somatic gonad likely contributes to meiotic initiation.

Early studies of RA activity proposed that RA diffuses through the fetal gonad in an A-P manner to produce an A-P wave of meiotic initiation (Figure 1 and Figure 2) (reviewed in [69,70]). While the mesonephros is attached to the gonad along its dorsal length, only the anterior mesonephric tubules are open and directly connected to the gonad (Figure 1) [71,72]. Thus, RA may diffuse from the mesonephros into the gonad via this anterior connection [8] (reviewed in [69]). Alternatively, some RA-producing cells may migrate from the anterior mesonephros into the anterior gonad (reviewed in [69]). Both scenarios could establish an A-P gradient that drives the observed wave of meiotic initiation. Consistent with this model, the *RARE*-*lacZ* reporter is detected in the fetal ovary in an A-P manner [8,9].

An A-P wave of *Dazl* expression precedes, and may also contribute to, the subsequent wave of meiotic initiation (Figure 1) [32]. On *Dazl* expression, germ cells acquire the ability to interpret RA as a meiosis-inducing factor [35] in an A-P manner (Figure 1) [32]. This wave of intrinsic germ cell competence may reinforce an RA gradient in inducing meiosis along the gonad. Alternatively, the A-P wave of intrinsic germ cell competence may drive the subsequent wave of meiotic initiation, independent of any differences in the local concentration of RA along the length of the gonad. Regardless, RA can induce *Dazl* expression in cultured PGC-like cells [73], which suggests an additional instructive role for RA in the development of germ cells in both the XX and XY-bearing cells, days prior to meiotic initiation.

### 2.5. Prevention of Meiotic Initiation in the Fetal Testis

In fetal testes, CYP26B1 degrades RA, thereby precluding the induction of *Stra8*, and preventing the initiation of meiosis (Figure 2) [7,8]. *Cyp26b1* is expressed in somatic cells of the developing testis (seminiferous) cords [7,8,74,75]. In *Cyp26b1*-deficient embryos, germ cells in the fetal testis express ectopic *Stra8* and initiate meiosis [8,76]. Thus, CYP26B1-expressing cells form a catabolic barrier that prevents RA, generated outside of the cords, from reaching the germ cells located within. The expression level of *Cyp26b1* in mouse fetal testes is maintained until E13.5, and reduced gradually thereafter [77]. The subsequent reduction of *Cyp26b1* may expose male germ cells to some RA, but male germ cells avoid a direct response, in part, through *Nanos2*, which prevents meiotic initiation in the fetal testis [77,78,79] (reviewed in [70,80,81]). The expression of *Nanos2*, which encodes a germ cell-specific RNA binding protein [82], is up-regulated from E13.5 onward and is restricted to the male germline [77,83]. In *Nanos2*-null embryos, male germ cells express low levels of *Stra8* and initiate ectopic meiosis at E14.5 [77], indicating that *Nanos2* operates subsequent to RA catabolism by *Cyp26b1* to prevent cells from initiating meiosis. The authors also reported that *Nanos2* inhibits meiosis, in part, by destabilizing *Dazl* and other down-stream targets (Figure 2) [79]. Thus, *Nanos2* is a cell-intrinsic factor that prevents the male germline from interpreting RA as a meiosis-inducing factor.

### 2.6. A Role for RA in the Ovary after Birth

After meiotic initiation, ovarian germ cells enter an extended meiotic prophase I, and begin differentiation as oocytes [36]. In mice, oocytes that progress through meiotic prophase I will arrest at the diplotene stage, also known as dictyate or germinal vesicle (GV) stage, around birth (reviewed in [84,85,86]). Shortly after birth, oocytes grow and differentiate independent of the chromosomal events of meiosis [87]. Meanwhile, oocytes organize the supporting somatic cells, called granulosa cells, to form follicles [88], which later undertake ovulation in response to hormonal stimulation. During and after puberty, groups of follicles will grow in size through both granulosa cell proliferation and the growth of the oocyte, which remain arrested at the GV stage (reviewed in [86]). Around the time of ovulation, full-grown GV stage oocytes resume meiosis, break down the nuclear envelope (GV breakdown), undergo meiotic progression, and arrest again at meiotic metaphase II (MII) until fertilization; the process from GV to MII stage is referred to as oocyte maturation, which is promoted by granulosa cells (reviewed in [84,85]).

Recent in vitro studies have proposed that both all-*trans* and 9-*cis* RA can act on granulosa cells to improve oocyte maturation in several mammals, including cows [89,90,91,92,93], goats [94], pigs [95], rats [96], and mice [97,98] (reviewed in [99,100]). RARs and RXRs are expressed in granulosa cells surrounding full-grown oocytes [96,101,102]. Supplementation of culture medium with all-*trans* or 9-*cis* RA induces granulosa cells to express genes that regulate differentiation and prevent apoptosis [90,92,93,94,103,104] (reviewed in [99]), suggesting that RA acts on granulosa cells to prevent their aberrant differentiation state and apoptosis. In vivo, *RARE*-*lacZ* signal is detected in granulosa cells of mouse ovarian follicles at 3 weeks of age, and increased after injection of a gonadotropic hormone [102], supporting a role for RA on these cells. Further in vivo studies are needed to determine whether RA is required by granulosa cells to support oocyte maturation in the ovary.

## 3. Development of Male Germ Cells after Birth

After birth, male germ cells differentiate into spermatogonia and initiate spermatogenesis, a process in which spermatogonial stem cells ultimately give rise to millions of haploid spermatozoa per day. Throughout spermatogenesis, several transitions occur in a strictly coordinated manner, including meiotic initiation, which is induced by periodic RA signaling, ensuring that spermatozoa are produced at a constant rate throughout reproductive life in males.

### 3.1. Organization of Spermatogenesis in the Postnatal and Adult Testis

**Figure 3:**
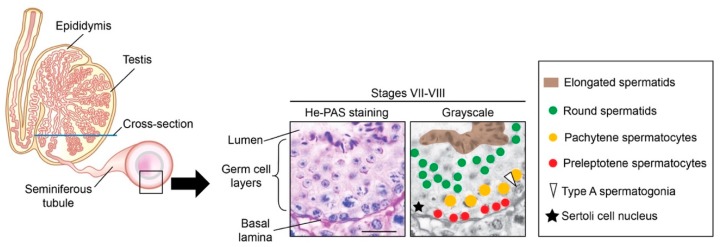
Structure of the mouse testis comprising seminiferous tubules. In any given tubule cross-section, one observes germ cells at different steps of their development into elongated spermatids. These germ cell types are concentrically layered; undifferentiated spermatogonia lie on the basal lamina of the tubule, and germ cells move toward the tubule lumen as they differentiate [106]. Germ cell differentiation is precisely timed; hence, particular steps of development are always found together in close physical proximity. Blue line indicates the orientation of testis cross-sections. A representative tubule cross-section in stage VII–VIII, stained with hematoxylin and periodic acid-Schiff (He-PAS), is shown with grayscale version. Star: Sertoli cell nucleus. White arrowhead: type spermatogonium. Dots: preleptotene (red) spermatocytes, pachytene spermatocytes (yellow), and step 7–8 round spermatids (green). Brown area: elongated spermatids. Scale bar = 30 μm.

In the fetal mouse testis, PGCs are enclosed by somatic cells as testis cords are formed between E12.5 to E14.0 (Figure 1) [14,23]. The germ cells present within the testis cords differ morphologically from migratory PGCs, and are called gonocytes [14,15]. Shortly after birth, the gonocytes, which are arrested in the G0/G1 phase [4,5], resume proliferation and migrate to the basement of the cords to give rise to type A spermatogonia (Figure 3) [14,15,105].

**Figure 4:**
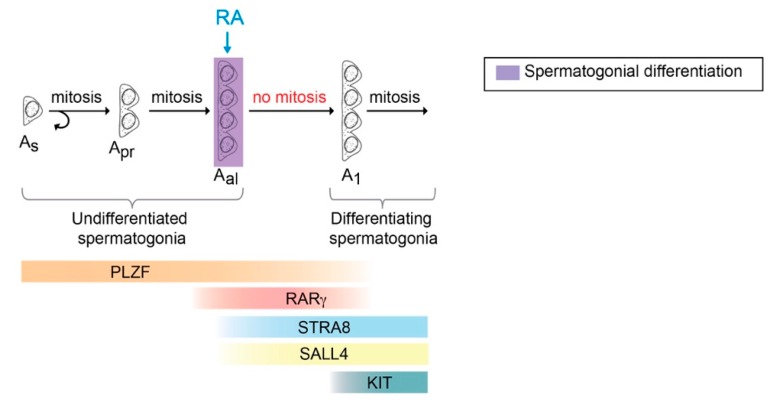
Multiplication of undifferentiated spermatogonia and spermatogonial differentiation. Upon division, the A_single_ (A_s_) spermatogonia can self-renew and produce two new singles or the daughter cells, A_paired_ (A_pr_) spermatogonia, remain connected by an intercellular bridge. The A_pr_ spermatogonia subsequently divide further into chains of 4, 8, or 16 cells, called A_aligned_ (A_al_) spermatogonia that undergo spermatogonial differentiation (purple) in response to RA. A_s_, A_pr_, and A_al_ spermatogonia are referred to as undifferentiated spermatogonia. After the spermatogonial differentiation, A_al_ spermatogonia transit into A_1_ differentiating spermatogonia without a mitotic division [114]. Expression patterns of PLZF, RARγ, STRA8, SALL4, and KIT are indicated as solid lines.

In mice, spermatogenesis begins with undifferentiated type A spermatogonia, which include the stem cells [107,108,109,110] (reviewed in [111]). Individual spermatogonial cells, known as A single (A_s_) spermatogonia, have traditionally been considered to encompass spermatogonial stem cells (Figure 4) [107,108,112]. Some of the A_s_ spermatogonia divide into paired A (A_pr_) spermatogonia, which are connected by an intercellular bridges. The A_pr_ spermatogonia subsequently divide further into extended chains of 4, 8, or 16 cells, called A_aligned_ (A_al_) spermatogonia. A_s_, A_pr_, and A_al_ spermatogonia are referred to as undifferentiated spermatogonia (Figure 4) (reviewed in [113]).

**Figure 5:**
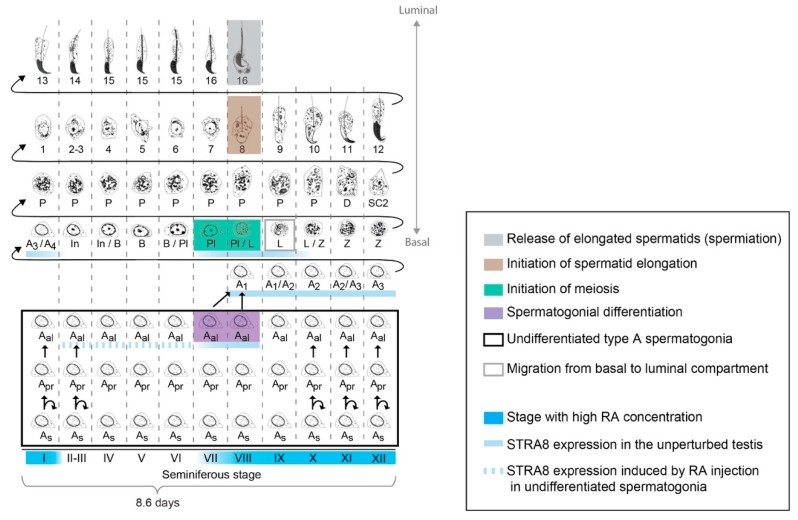
Diagram of mouse spermatogenesis. Oakberg [106] identified 12 distinct cellular associations, called seminiferous stages I–XII. It takes 8.6 days for a section of seminiferous tubule, and the germ cells contained within, to cycle through all 12 stages [122]. Four turns of this seminiferous cycle are required for a germ cell to progress from undifferentiated spermatogonium to spermatozoon. A_s_, A_pr_, and A_al_: A_single_, A_paired_, and A_aligned_ spermatogonia. A_1_–A_4_: A_1_–A_4_ differentiating spermatogonia. In, and B: intermediate and type B spermatogonia. Pl, L, Z, P, D, and SC2: preleptotene, leptotene, zygotene, pachytene, diplotene, and secondary spermatocytes. Steps 1–16: steps in spermatid differentiation. Purple: germ cells undergoing spermatogonial differentiation; green: meiotic initiation; brown: initiation of spermatid elongation; gray: release of elongated spermatids. Black box: population of undifferentiated spermatogonia. Gray box: the leptotene spermatocytes undergoing migration of basal to luminal compartment [123]. Dark blue: stage with high RA concentration. Light blue line: STRA8 expression in the unperturbed testis. Dashed light blue line: STRA8 expression induced by RA injection in undifferentiated spermatogonia. (After RA injection, undifferentiated A_al_ spermatogonia in stages II–VI precociously express STRA8 [124]).

Undifferentiated spermatogonia periodically commit to differentiation, in the form of an A_al_-to-A_1_ transition, to become differentiating spermatogonia, which encompass A_1_, A_2_, A_3_, A_4_, intermediate and B spermatogonia (Figure 4 and Figure 5) (reviewed in [114,115]). During differentiation, spermatogonia lose the capacity for self-renewal [116], accelerate their cell cycle [117], and undertake six mitotic divisions in mice [118]. Germ cells then differentiate to spermatocytes and undergo meiotic initiation (Figure 5) [18,36]. DNA replication and two cell divisions follow, resulting in the formation of haploid, round spermatids, which elongate their nucleus and cytoplasm to become elongated spermatids. Finally, these spermatids are released into the lumen of the seminiferous epithelium, whereupon they are referred to as spermatozoa (Figure 3 and Figure 5) (reviewed in [119]). These layered generations of germ cells are embedded in and supported by somatic Sertoli cells that supply factors essential for spermatogenesis (Figure 3) (reviewed in [120,121]).

Within cross-sections of the seminiferous epithelium, stereotypical collections or associations of germ cells occur at various steps of differentiation (Figure 3 and Figure 5). The precise coordination of these steps is called the “cycle of the seminiferous epithelium” (or “seminiferous cycle”). In mice, the seminiferous cycle has been subdivided into 12 distinct cellular associations, known as seminiferous (epithelial) stages I to XII [106]. During spermatogenesis, four transitions direct key phases of germ cell development: (i) differentiation of spermatogonia, (ii) meiotic initiation, (iii) initiation of spermatid elongation, and (iv) the release of elongated spermatids into the lumen of seminiferous tubules (spermiation) (Figure 5). These four transitions are precisely coordinated in time and space, each occurring in stages VII and VIII of the seminiferous epithelium (Figure 5) [106] (reviewed in [16,111]). The close physical and temporal proximity of each of these transitions, occurring cyclically, with an 8.6-d periodicity in mice [122], suggests a strict coordination. The intimate proximity of each of these transitions is largely conserved in other mammals, including humans [125], rats [112,126], hamsters [127], and rams [127].

### 3.2. Regulation of Spermatogenesis by Vitamin A and RA

A central role for RA in mammalian spermatogenesis was first described in 1925, when rodents fed a vitamin A-deficient (VAD) diet were found to be sterile [128,129,130] (reviewed in [131,132]). In VAD mice and rats, most germ cells arrest as undifferentiated spermatogonia [133,134,135,136,137]. In VAD rat testes, some germ cells arrest just prior to meiosis, as preleptotene spermatocytes [17,136,138]. When VAD animals are given an injection of all-*trans* RA, or vitamin A, the arrested spermatogonia undertake differentiation [17,135,137], and the arrested preleptotene spermatocytes initiate meiosis [17]. Further, mice treated daily with WIN18,446—which inhibits the retinaldehyde dehydrogenases (ALDH1A1-3) and thereby prevents local RA production [139,140]—exhibited blocks in both spermatogonial differentiation and meiotic initiation [124,141,142]. Thus, in males, both these premeiotic transitions—spermatogonial differentiation and meiotic initiation—require RA.

### 3.3. The Role of RA and Stra8 at Spermatogonial Differentiation and Meiotic Initiation

*Stra8*, which is required for meiotic initiation, also promotes (but is not strictly required for) spermatogonial differentiation [124]. In the postnatal mouse testis, the STRA8 protein is detected in spermatogonia as early as postnatal day 2 (P2) [47,143], when the first evidence of spermatogonial differentiation occurs [144]. In the adult testis, STRA8 is expressed at spermatogonial differentiation of A_al_ spermatogonia, and in preleptotene spermatocytes at meiotic initiation; both occur during stages VII–VIII (Figure 4 and Figure 5) [124,145,146]. In mice lacking *Stra8*, undifferentiated spermatogonia accumulate in unusually high numbers as early as P10 [124], and the remaining germ cells arrest just prior to meiosis, as preleptotene spermatocytes [18,36]. Thus, RA acts instructively, and at least in part through STRA8, at spermatogonial differentiation, distinct from its critical function in meiotic initiation [124].

Unlike RA deficiency, genetic ablation of *Stra8* does not preclude spermatogonial differentiation [124], indicating that RA has additional effects, aside from inducing *Stra8* expression, at this transition. Culture experiments [48,147] indicate that treatment of undifferentiated spermatogonia with RA stimulates the expression of *Stra8* and of *Kit*, a marker of spermatogonial differentiation [148,149,150]. In vivo, *Kit* expression is low in undifferentiated spermatogonia due, in part, to the action of PLZF (also known as ZBTB16). In germ cells, PLZF maintains spermatogonia in an undifferentiated state [151,152] by binding the *Kit* promoter and repressing its expression [153] (Figure 4). At spermatogonial differentiation, RA induces the expression of its target gene *Sall4*, which sequesters PLZF from the *Kit* promoter, thereby increasing *Kit* expression (Figure 4) [154,155] (reviewed in [156]). RA has also been found to activate the PI3K-AKT-mTOR signaling cascade in a non-genomic manner, stimulating the translation of *Kit* mRNA [157] (reviewed in [158]). Thus, RA may induce spermatogonial differentiation via several independent pathways, including *Stra8*, *Sall4*, and *Kit*.

During spermatogonial differentiation, RA acts directly on germ cells through RARs. Undifferentiated spermatogonia express several RARs (Figure 4) [159,160], and simultaneous ablation of both RARγ and RARα in germ cells impairs spermatogonial differentiation [159] (reviewed in [145]). Additional targets of RA could be activated indirectly, by the action of RA on the Sertoli cells, as RA signaling via RARα in Sertoli cells is critical for the first round of spermatogonial differentiation [63], and for the differentiation of Sertoli cells at puberty [161]. 

### 3.4. Role of RA at the Initiation of Spermatid Elongation and Spermiation

In the 1980s, Huang and Marshall [162] suggested that vitamin A deficiency may delay spermiation. Moreover, ablation of RARs or RA-synthesizing enzymes (in germ cells and/or Sertoli cells) causes a variety of defects in both meiotic and postmeiotic transitions, including spermiation [63,163,164,165,166,167,168]. A recent study has shown that RA plays primary roles at two postmeiotic transitions; the initiation of spermatid elongation and spermiation (Figure 5) [19]. After injection of the inhibitor WIN18,446, both spermatid elongation and spermiation were delayed, and conversely, a single injection of RA was sufficient to precociously induce both these transitions.

It remains to be determined whether the requirement for RA at these two post-meiotic transitions is due to the direct action of RA on germ cells, or occurs indirectly, via Sertoli cells. RARs and RXRs are expressed specifically in round spermatids in stages VII and VIII [60], suggesting that RA may act directly on round spermatids to initiate elongation. Indirect RA signaling, via RARs/RXRs in Sertoli cells [60], may also contribute to this process. RA is likely to regulate the release of elongated spermatids indirectly, via Sertoli cells, as these spermatids are thought to be transcriptionally silent (reviewed in [169]). By identifying RA functions in post-meiotic cells, future studies may resolve the mechanism by which RA regulates each of these two postmeiotic transitions.

### 3.5. Source of RA in the Postnatal and Adult Testis

In postnatal and adult testes, RA-degrading enzymes (*Cyp26a1*, *Cyp26b1*, and *Cyp26c1*) are expressed by peritubular myoid cells that surround the seminiferous tubules [60]. These peritubular myoid cells form a catabolic barrier that prevents RA generated outside of the seminiferous epithelium from reaching the enclosed germ cells [156]. In the seminiferous tubule, RA is produced by two different cellular sources, Sertoli cells and germ cells. Sertoli cells express an RA-synthesizing enzyme, *Aldh1a1* [60,170]. Another RA synthesizing enzyme, *Aldh1a2*, is expressed in pachytene and diplotene spermatocytes from stages VII through XII [60,170]. Indeed, direct quantitation of RA levels confirms that both *Aldh1a1*-expressing Sertoli cells and the *Aldh1a2*-expressing germ cells contribute to the total production of RA from circulating retinol [19,171].

The RA produced by Sertoli cells is required for spermatogonial differentiation. Sertoli cell-specific ablation of *Aldh1a1*-*3* causes a complete arrest at the first round of spermatogonial differentiation in postnatal mice [63]. In the unperturbed testis, RA from Sertoli cells contributes functionally to both spermatogonial differentiation and meiotic initiation [19]. Recent studies have addressed the question of whether RA produced by pachytene spermatocytes is required for spermatogenesis [19,171,172]. Chemical or genetic depletion of pachytene spermatocytes in adult testes results in delays to both the elongation of the round spermatids and spermiation, but not to spermatogonial differentiation or meiotic initiation [19]. Germ cell-specific ablation of *Aldh1a1*-*3* delays the first round of postnatal spermatogenesis, but these animals show complete spermatogenesis in adult testes at 8 to 10 weeks [171]. The simplest interpretation of these findings is that, in the unperturbed testis, pachytene spermatocytes work collaboratively with Sertoli cells to produce RA levels for the four transitions.

In mice with a Sertoli cell-specific deletion of *Aldh1a1*-*3*, the arrest at the first round of spermatogonial differentiation can be rescued by RA injection, as all germ cell cohorts are subsequently observed [63]. Conversely, after injection of RA at 4 weeks of age, Sertoli cell-specific *Aldh1a1*-*3*-deficient adults displayed abnormalities in spermiation at 24 weeks of age [63], suggesting that RA from Sertoli cells contributes modestly to this process. Moreover, the level of RA required for spermatogonial differentiation is higher than that required for meiotic initiation [171,172], indicating that each transition is sensitive to the local level of RA. Because the postmeiotic transitions are most sensitive following depletion of RA [19], the postmeiotic transitions may require a higher concentration of RA, from both Sertoli cells and pachytene spermatocytes, than the premeiotic transitions.

When mice lacking *Aldh1a1*-*3* in both Sertoli cells and germ cells are given a single RA injection at P3, some germ cells immediately undergo spermatogonial differentiation and later initiate meiosis (with STRA8 expression) seven days after the injection [171]. Based on this observation, Teletin et al. [171] hypothesized that RA is dispensable for meiotic initiation. However, after a single injection of exogenous RA to postnatal mice, increased levels of RA in the testis are maintained for more than seven days, even under the daily treatment with WIN18,446, which inhibits endogenous RA production [172]. Given that meiotic initiation can be induced by a low threshold of RA [171,172], the injected RA remaining in the seminiferous tubule may be sufficient to induce meiotic initiation in postnatal mice.

### 3.6. Periodicity of Spermatogenesis and RA Levels

In the unperturbed testis, STRA8 is periodically expressed in spermatogonia and is present during the majority of the seminiferous cycle. Specifically, STRA8 is rarely expressed in stages II–VI (before the four transitions), then increases rapidly in stages VII–VIII (during transitions), and remains high thereafter in stages IX–I (Figure 5) [124,145,146]. The expression of STRA8 reflects the presence of RA; when RA levels are increased by injecting RA, or decreased by injecting WIN18,446, STRA8 expression is immediately induced or absent, respectively, in all seminiferous stages, as judged by immunostaining [124]. In good agreement with STRA8 expression, RA concentrations change periodically in the seminiferous tubule [124,146]; absolute quantification of RA levels has found that RA levels are low in stages II–VI, rise in stages VII–VIII, and remain high until stages XII/I (Figure 5) [124]. The expression of RA-metabolizing enzymes may help to explain how this periodicity of RA concentration is established in the adult testis. *Aldh1a1* transcripts are present in stages I–VIII in Sertoli cells [156,170], and *Aldh1a2* transcripts peak in late pachytene and diplotene spermatocytes in stages VII–XII [60,170]. Thus, Sertoli cell production of RA may precede the germ cell production of RA in each cycle of the seminiferous epithelium. In contrast, the expression of RA storage enzymes, *Lrat* and *Adfp*, which function to reduce local RA levels, are detected in stages I–VI/VII [60,170]. Thus, RA concentration in stages II-VI might be kept low, even in the presence of Aldh1a1. Moreover, the CYP26 family of enzymes, which are expressed in Sertoli cells [60,170,173,174], may catabolize RA to maintain tight control of the seminiferous milieu.

### 3.7. Competence of Germ Cells for Spermatogonial Differentiation

Despite the persistently elevated RA levels in stages IX–I, spermatogonial differentiation is not observed in these stages (Figure 5). Early undifferentiated A_s_ and A_pr_ spermatogonia (found at all stages) and undifferentiated A_al_ spermatogonia in stages IX–I are unable to express STRA8 in response to RA injection, and do not undergo differentiation (Figure 5) [124]. In the presence of RA, these undifferentiated spermatogonia instead undertake self-renewal and proliferation, which prevents the pool of undifferentiated spermatogonia from being irreversibly depleted. This competence or incompetence for spermatogonial differentiation cannot simply be explained by the expression of RARs, as these receptors are broadly expressed across the seminiferous cycle [60,159,160]. Instead, competence for spermatogonial differentiation is more closely correlated with the proliferative activity of the cells. Specifically, undifferentiated spermatogonia in stages II–VIII, which are competent for differentiation [124], are arrested in the G0/G1 phase of the cell cycle, whereas undifferentiated spermatogonia in stages IX–I are actively proliferating (Figure 5) [107,117]. Further studies are needed to identify the mechanisms that confer competence for spermatogonia to undergo differentiation.

## 4. Summary and Perspectives

Several lines of evidence support a critical role for RA in directing meiotic initiation in the fetal ovary, and for critical transitions of adult spermatogenesis, including meiotic initiation. In development, embryonic germ cells acquire the competence to initiate meiosis in response to RA. Male germ cells, which escape from RA-induced meiotic initiation in the fetal testis by the catabolism of RA, develop first as undifferentiated spermatogonia, which later acquire competence for spermatogonial differentiation. Male germ cells subsequently acquire competence for meiotic initiation (and possibly initiation of spermatid elongation). These distinct competencies to respond to RA must be strictly regulated. After RA injection, undifferentiated spermatogonia are not competent to initiate meiosis directly; instead, the undifferentiated spermatogonia begin a program of spermatogonial differentiation, followed by six mitotic cell divisions [124]. Further studies of these distinct competencies will help our understanding of the basic mechanisms that govern germ cell development and advance assisted reproduction technologies, such as in vitro gamete production [175,176,177,178].
